# Production of carbon-11 for PET preclinical imaging using a high-repetition rate laser-driven proton source

**DOI:** 10.1038/s41598-024-61540-2

**Published:** 2024-05-20

**Authors:** Juan Peñas, Aarón Alejo, Adrián Bembibre, Jon Imanol Apiñaniz, Enrique García-García, Carlos Guerrero, José Luis Henares, Irene Hernández-Palmero, Cruz Méndez, María Ángeles Millán-Callado, Pilar Puyuelo-Valdés, Michael Seimetz, José Benlliure

**Affiliations:** 1 Instituto Galego de Física de Altas Enerxías (IGFAE), Universidade de Santiago de Compostela, 15782 Santiago de Compostela, Spain; 2 Centro de Láseres Pulsados (CLPU), 37185 Salamanca, Spain; 3 Dpto. Física Atómica, Molecular y Nuclear (FAMN), Universidad de Sevilla, 41012 Sevilla, Spain; 4 Centro Nacional de Aceleradores (CNA) (US-Junta de Andalucía – CSIC), 41092 Sevilla, Spain; 5 Instituto de Instrumentación para Imagen Molecular (I3M), CSIC – Universitat Politècnica de València, 46022 Valencia, Spain; 6 Present Address: Instituto de Física Corpuscular (CSIC-UV), 46071 Valencia, Spain

**Keywords:** Physics, Nuclear physics, Experimental nuclear physics, Lasers, LEDs and light sources, Diagnostic markers

## Abstract

Most advanced medical imaging techniques, such as positron-emission tomography (PET), require tracers that are produced in conventional particle accelerators. This paper focuses on the evaluation of a potential alternative technology based on laser-driven ion acceleration for the production of radioisotopes for PET imaging. We report for the first time the use of a high-repetition rate, ultra-intense laser system for the production of carbon-11 in multi-shot operation. Proton bunches with energies up to 10–14 MeV were systematically accelerated in long series at pulse rates between 0.1 and 1 Hz using a PW-class laser. These protons were used to activate a boron target via the $^{11}$B(p,n)$^{11}$C nuclear reaction. A peak activity of 234 kBq was obtained in multi-shot operation with laser pulses with an energy of 25 J. Significant carbon-11 production was also achieved for lower pulse energies. The experimental carbon-11 activities measured in this work are comparable to the levels required for preclinical PET, which would be feasible by operating at the repetition rate of current state-of-the-art technology (10 Hz). The scalability of next-generation laser-driven accelerators in terms of this parameter for sustained operation over time could increase these overall levels into the clinical PET range.

Since their inception, single-photon emission computed tomography (SPECT) and positron emission tomography (PET) imaging techniques have undergone a constant development and are now considered well-established procedures in nuclear medicine. One major advantage of these techniques over other imaging methods is that they allow *in vivo* visualization of pathological and physiological processes^1^, as radiotracer distribution and metabolism can be observed in real time. The overall demand for radioisotopes used in medical applications has recently increased due to advancements in both SPECT and PET^2,3^. These radioisotopes are primarily produced at specialized nuclear reactors and conventional radio-frequency (RF) accelerators, which are responsible for regional or even national supply. The elevated costs associated with these production centers, particularly in terms of radiation shielding requirements, necessitate the production of large, single doses to be distributed to as many clinics, hospitals, or research centers as possible. An example is the case for $\beta ^+$ emitters used in PET imaging, for which commercial production is typically restricted to fluorine-18: due to its long half-life ($\sim$110 minutes), the total time required for its production, processing, and distribution can be managed at the expense of producing higher doses.

On the other hand, short-lived radioisotopes utilized in PET imaging such as carbon-11 ($\sim$20 min), nitrogen-13 ($\sim$10 min), or oxygen-15 ($\sim$2 min) are increasingly in demand due to their ability to provide high-resolution imaging with minimal exposure time for the patient. Given their short half-life, higher count rates can also be achieved at moderate activity levels. The particular case of carbon-11 ($^{11}$C) is of special interest as it can be coupled to a large variety of biochemical radiotracers that target specific organs or tissues^4,5^, due to its well-known chemistry. Its half-life is also sufficient for more complex processing techniques. $^{11}$C has been extensively used in the diagnosis of neurodegenerative diseases such as Alzheimer’s or Parkinson’s, as well as several types of cancer^4^. However, the rapid decay rate of short-lived radioisotopes requires immediate use, hence the production of limited doses is generally beyond the scope of conventional production facilities.

In this context, ultra-short, ultra-intense lasers have been considered as a cost-effective alternative to conventional methods for the on-demand production of single doses of short-lived radioisotopes^6,7^. Through the well-understood Target Normal Sheath Acceleration (TNSA) mechanism^7,8^, ultra-intense laser pulses (*I* > 10$^{18}$ W/cm$^2$) impinging on solid micron-thickness target foils can efficiently accelerate ions up to several MeV. Due to the micron-scale size of the laser-solid interaction, the shielding requirements for these facilities are only restricted to the immediate vicinity of the interaction point. These conditions significantly reduce their size and cost compared to RF accelerators. Similar to conventional facilities, ions from a laser-driven source can induce nuclear reactions upon reaching a secondary target, resulting in the production of specific radioisotopes through target material activation. One notable advantage of this technology is the potential of producing specific doses of a wide variety of radioisotopes depending on the irradiation time and the target and laser parameters. Such fundamental flexibility could allow for effective on-demand dose production^9^.

The unique properties of laser-accelerated ions, such as high brightness, low emittance, and a broad, Maxwellian-like energy spectrum make them suitable for radioisotope production. All these properties are illustrated in Fig. 1, which depicts the cross-section of some proton-induced nuclear reactions for the production of radioisotopes relevant in PET medical imaging. The blue solid line corresponds to an example of a proton energy spectrum measured within this work (see *Results and discussion* section). A significant part of the spectrum is above the reaction thresholds (2–4 MeV) and reaches the maximum of the given excitation functions. Hence, the wide spectrum of laser-accelerated protons is not a limitation for the production of radioisotopes. The fact that protons are the dominant species in laser-driven sources is another favorable factor, as most of the common production reactions are induced by light particles such as protons or deuterons. Therefore, due to both physical and economical reasoning, the use of laser-driven ion sources for radioisotope production offers significant benefits in terms of cost-effectiveness, efficiency, and accessibility.

**Figure 1:**
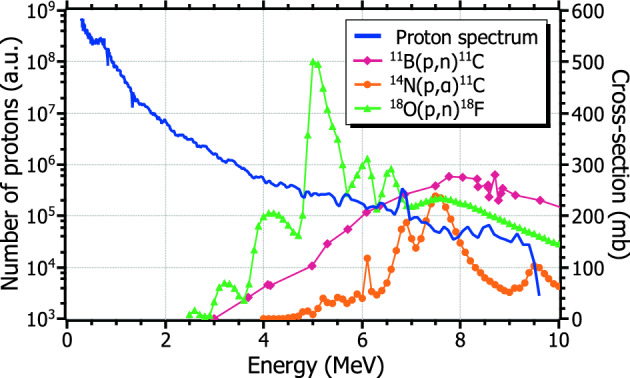
Cross-sections of some proton-induced production reactions of radioisotopes of interest in PET (right axis), along with one of the experimental proton spectra measured within this work (blue curve, left axis). Cross-section data retrieved from M.L. Firouzbakht *et al*.^10^ and the IAEA database^11^.

Typically, the activity levels currently handled in PET preclinical imaging are in the range of 10–30 MBq^12,13^. For clinical imaging, these requirements escalate up to several hundreds of MBq or even a few GBq^14,15^. As the efficiency in both the imaging procedures and the scanners improves year-to-year, the minimum doses for practical use tend to decrease and become more accessible to alternative production techniques^16–18^. These achievements are also directed towards the reduction of the effective doses provided to the patient. Due to this, the growing interest in laser-driven acceleration for radioisotope production has led to numerous studies of the achievable activity in terms of laser and target parameters for a wide variety of laser systems^19^. The action plan of current laser facilities can be separated in two different approaches for achieving practical activity levels. On one hand, high-energy laser systems focus on reaching laser pulses of huge energy ($\ge$100 J per pulse) that are able to produce a large number of nuclear reactions in a single shot^20–22^. Their repetition rate, however, is limited to several minutes between shots. On the other hand, table-top, high-repetition rate laser systems ($\sim$1 J per pulse) would be able to produce moderate activities per shot but maintaining an acceptable production rate in the multi-Hz scale over long operation times^23–25^.

**Table 1:** State-of-the-art in laser-driven $^{11}$C production for PET imaging.

References	Laser energy (J)	Pulse duration (fs)	Carbon-11 activity (kBq)
Spencer *et al*.^21^	120	900–1200	200
Ledingham *et al.* ^22^	300	750	6000
Fritzler *et al*.^23^	1	40	1.2
Tayyab *et al*.^24^	2.4	25	5.2

The indicated activities correspond to single-shot experimental measurements from various recent publications. The first two rows belong to high-energy laser facilities, while the other two were measured at compact, table-top systems. The laser pulse energy and duration are also indicated.

In regard to the current state-of-the-art of radioisotope production from laser-driven sources, all the published data so far belong to single-shot operation. Table 1 summarizes some of the most relevant publications where the production of $^{11}$C through proton-induced reactions is explored, which aligns with the main objective of this work. Among the published studies, the validation of the technology as a potential approach for general radioisotope production has been performed at high-energy laser systems^21,22^. Despite the high activity per shot obtained, between hundreds of kBq and several MBq, the production of radioisotopes at these large-scale lasers is mainly limited by their pulse repetition rate and technologically restricted to single-shot operation.

In order to produce useful doses for PET imaging, high-repetition rate lasers could represent a more suitable option. Multi-shot operation, which has been only explored through simulations and extrapolations so far^25,26^, is required to compensate for the lower activities per shot, of a few kBq. The requirements of such operation entail the accumulation of a large number of shots (1000s) over long operation times at high-repetition rates (multi-Hz). These implications lead to a series of technological constraints in terms of shot-to-shot stability and repeatability in order to maximize the conversion efficiency and to allow for sufficiently long operation times. Namely, the refreshment of the material of the acceleration targets, as they are destroyed by the laser pulses at each shot, and the required micron-order positioning accuracy of their surface at the focal spot of the laser. The development of a multi-shot target system capable of operating during long irradiation series while solving these constraints is thus mandatory for radioisotope production.

Through the procedure presented in this work, the experimental viability of high-repetition rate laser systems as an approach to radioisotope production has been explored. In particular, the achievable activity in the context of the minimum required for PET preclinical medical imaging. As a first step, in this paper we report the systematic acceleration of MeV-order protons at high repetition rates in a PW-class laser system. To the best of our knowledge, such operation was achieved for the first time for long irradiation series of more than a hundred of shots. The production of $^{11}$C was then induced through the $^{11}$B(p,n)$^{11}$C nuclear reaction by the irradiation of a boron target with protons from the laser-driven source in multi-shot operation, i.e., by accumulating a certain amount of shots over a fixed time. This performance also constitutes a novel result in the context of laser-driven radioisotope production. The absolute activity of the target after the irradiation was determined and related to the activity levels required for PET.

## Experimental set-up

**Figure 2:**
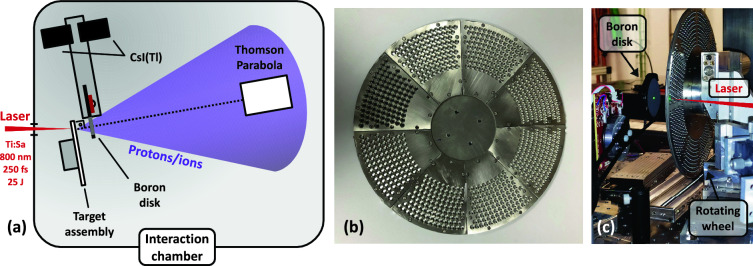
(**a**) Schematic of the experimental set-up. (**b**) Image of the target wheel used in the experiment. (**c**) Image depicting the boron disk placed at the proton flight path, behind the acceleration target. The green spot at the center of the boron target corresponds to an alignment visible laser, coupled to the flight path of the accelerated ions.

The experiment was carried out at the PW-class VEGA-3 line at the Centro de Láseres Pulsados (CLPU)^27^ in Salamanca, Spain. This laser line delivered pulses of up to 25 J before compressor at a maximum repetition rate of 1 Hz. Within the experiment, the duration of the laser pulses was 250 fs. The experimental set-up inside the interaction chamber is depicted in Fig. 2a. Laser pulses with *p*-polarisation were focused by means of an *f*/11 Off-Axis Parabola (OAP) at an incidence angle of 10$^{\circ }$ onto 7 µm-thick aluminium foils with a spot size of around 20 µm in diameter.

The target assembly used for ion acceleration within the experiment was an adaptation of a previous design presented in Ref.^28^, based on a wheel-like holder hosting thin foils for operation up to 10 Hz. The design of this target assembly allows for the refreshment of the target material before the arrival of each laser shot while placing its surface at the laser focus with micron accuracy. An image of the adapted wheel for this experiment can be seen in Fig. 2b. The wheel-like holder consists of a stainless steel base disk of 244 mm-diameter and eight sector plates that host the target foils in between. Each sector is drilled with a matrix of 101 holes of 2.3 mm-diameter arranged along nine arcs at different radii. Thus, the total number of targets of this holder version is 808. The pattern of individual holes was chosen in order to isolate each impact position and prevent possible effects of the laser pulses impinging on adjacent ones, given the high energy per pulse at the laser beamline. The center-to-center distance between holes and between radii was set to 5.5 mm, enough for the maximum repetition rate of 1 Hz at VEGA-3. The target wheel was attached to a set of three high-precision motorized stages for positioning the target foils at the laser focus, following an effective procedure that ensures a few-micron accuracy and a high repeatability while operating at high repetition rates^28^.

The characterization of the laser-accelerated ion beam, prior to the radioisotope production series, was performed using a Thomson parabola spectrometer (TPS), placed at 72 cm from the target system and along the normal direction of the Al target foils (see Fig. 2a). By means of this device, the energy spectra of the accelerated protons could be measured on a shot-to-shot basis, as well as the species composition of the ion bunches.

For the $^{11}$C production series, a boron disk (99% purity) of 2.54 cm diameter and 2 mm-thickness was utilized to induce nuclear activation through the $^{11}$B(p,n)$^{11}$C reaction. The disk was supported by a paddle-like holder placed on a rotating stage, in turn mounted on a linear motorized platform. The diagnosis device consisted of a pair of thallium-doped cesium iodine (CsI(Tl)) radiation detectors, situated face to face far from the proton flight path, that served to correlate the back-to-back photons from the annihilation of the positrons emitted in the $\beta ^+$ decay of the $^{11}$C nuclei. The combination of the linear movement and the rotation of the paddle-like holder allowed for placing the boron disk under the path of the proton beam, for nuclear activation during the laser irradiation series, or between the two CsI(Tl) detectors, for the activity characterization. An image of the former position can be seen in Fig. 2c. The production of $^{11}$C in the boron target was measured in temporal coincidence to discriminate events corresponding to positron annihilation. The detectors were calibrated to obtain absolute values of the total induced activity. Details of both the measurement procedure and the calibration of the detectors efficiency can be found in the *Methods* section.

## Results and discussion

### The proton source

**Figure 3:**
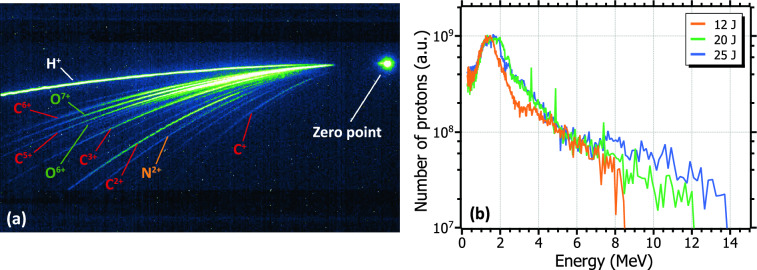
(**a**) TPS trace showing the the species composition of the ion beam accelerated by a 25 J laser pulse. (**b**) Proton spectra retrieved from the TPS at various laser energies: 12 J (orange), 20 J (green), and 25 J (blue).

The species composition of the laser-accelerated ion beam is depicted in Fig. 3a, showing the traces due to protons (H$^+$) and a collection of carbon ($^{12}$C), nitrogen ($^{14}$N), and oxygen ($^{16}$O) ions with different charge states. Laser-accelerated ions are characterized by a wide, Maxwellian-like energy spectrum with a sharp cut-off at the higher energy limit. Fig. 3b depicts some examples of the energy spectra extracted from the proton traces registered at the TPS. In order to correlate the proton cut-off with the laser energy, and ultimately to study the dependence of the produced $^{11}$C activity on the same parameter, measurements were performed at several laser pulse energies. Specifically, three laser energies were taken into consideration: 12 J, 20 J, and 25 J (orange, green, and blue curves in Fig. 3b, respectively). The criteria for determining the cut-off energy of a given proton trace was the more energetic signal value—closest to the zero point— distinguishable from the average background. As can be seen in the figure and reported in literature^29,30^, increasing the laser energy leads to an increase in the cut-off energy. Proton cut-off energies between 10 and 14 MeV were systematically observed for the maximum laser pulse energy. This variation in the value of the maximum energy was likely produced due to differences in the laser parameters and the focal spot quality on different measuring days.

**Figure 4:**
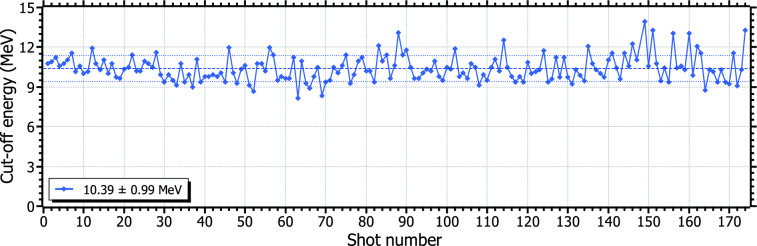
Multi-shot stability of the proton cut-off energy for a series at 25 J-pulse energy at 0.1 Hz, as measured by the TPS. The dashed lines indicate the mean value and the standard deviation.

Several multi-shot series of more than 101 shots—an entire sector of the target wheel— were performed at various repetition rates between 0.1 and 1 Hz. The measured proton cut-off energies within the same series exhibit high stability and a high repeatability within the same day. The standard deviation is below the 10% in most of the series in multi-shot operation, regardless of the pulse energy or the repetition rate, and may be attributed to shot-to-shot fluctuations of the laser system. Fig. 4 shows the proton cut-off energy, retrieved from the analysis of the TPS traces, for a single series of 174 shots at 25 J-pulse energy and 0.1 Hz. By the time of writing, this performance constitutes a novel achievement concerning long, sustained operation at high repetition rates using a PW-class laser system.

The high shot-to-shot stability of the results shown in this section may be promising for certain applications of laser-driven accelerators, such as radioisotope production, that require a large number of accumulated shots. In contrast to high-energy laser systems where huge amounts of nuclei are activated on a single laser shot, high-repetition rate lasers rely on a sustained operation over time to produce sufficient doses. Moreover, increasing the pulse rate would lead to achieving a certain activity on a shorter time, and thus reduce the losses due to the radioactive decay of the produced radioisotopes. Present compact, table-top laser systems are in principle able to operate at pulse rates in the multi-Hz scale, given their lower energy per pulse. However, due to actual technical constraints in most of current target system designs, e.g., the accurate positioning of the targets, practical operation is restrained to almost 1 Hz^31,32^. These restrictions are even greater for PW-class lasers. While the operation rate was limited to 0.1 Hz at the maximum laser pulse energy during this experiment (see *Methods* section), further optimization of the system would allow for operating at 1 Hz or higher repetition rates at such pulse energy.

### Production of $^{11}$C

The production of $^{11}$C through the aforementioned reaction was successfully achieved by the irradiation of the boron target. Several series in multi-shot operation were performed at different laser pulse energies: 12, 20, and 25 J. Each series took place on different days so the presence of $^{11}$C was completely cleared from the boron disk before the next irradiation. Table 2 summarizes the experimental parameters of each irradiation. The measurement of the activity of the boron target was performed between 10 and 20 minutes after each irradiation. For each series, the total induced activity of the sample was determined from the temporal coincidence measurement of the two back-to-back photons produced in the annihilation of the emitted positrons, measuring at time intervals ranging from 1 to 5 minutes over two to three $^{11}$C half-life periods (20.23 min).

**Table 2:** Experimental parameters of the multi-shot series for nuclear activation and $^{11}$C production results on each of the series.

Laser energy (J)	Repetition rate (Hz)	Number of shots (−)	Prod. per shot (10$^6$ nuclei)	Activity per shot (kBq)	Total activity (kBq)
12	0.2	40	3.2	1.8	68.6
20	0.1	10	5.7	3.2	31.5
25	0.1	20	21.7	12.4	233.8

The estimated uncertainty for the reported production and activity values is less than 1.5%.

**Figure 5:**
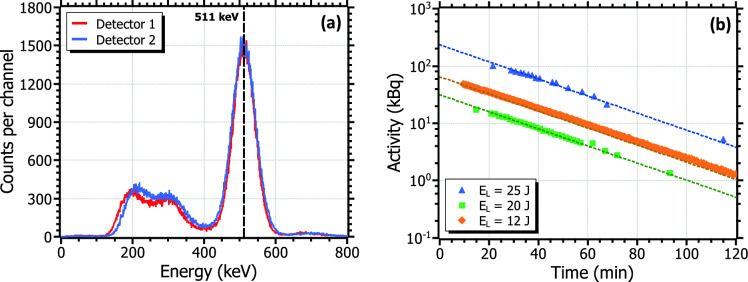
(**a**) Measured $^{11}$C spectra at each detector after the laser irradiation, showing the characteristic photopeak at 511 keV. (**b**) $^{11}$C decay curves after the laser irradiation for each of the experimental series (see Table 2 for details about the irradiations). The dashed lines are fits to a simple exponential decay function.

The energy spectrum obtained by each of the two detectors in one of these series is shown in Fig. 5a. The 511 keV line from the positron annihilation can be observed in both signals. The instantaneous activity at a certain time after the end of the irradiation, taking into account the detection efficiency of the system, was obtained via Eq. 2 (see *Methods* section). For each laser pulse energy, the activity curve was obtained by fitting the experimental data to Eq. 3, as depicted in Fig. 5b. A half-life of 20.23 ± 0.01 min was obtained from the fits, thus demonstrating, along with the 511 keV photopeak of the energy spectra, the production of $^{11}$C. The peak activity, achieved immediately after the last shot of each irradiation series, could be directly obtained from Eq. 3. From this value, the activity per shot was calculated using Eq. 4 and the tabulated disintegration constant of $^{11}$C, $\lambda _{^{11}C}$ = 5.7$\cdot$10$^{-4}$ s$^{-1}$. Both the peak and per shot activities obtained at each of the irradiation series are included in Table 2. The highest activity achieved during the experiment was 234 kBq, obtained after 20 shots at 0.1 Hz-repetition rate for a pulse energy of 25 J, which corresponds to an average activity per shot of 12.4 kBq. Regarding the other two series at lower pulse energies, peak activities of between 30 and 80 kBq were measured, for activities per shot of around 2–3 kBq. Albeit longer irradiations were technically feasible, the radioprotection requirements within the experiment limited the series to a few tens of shots only.

**Figure 6:**
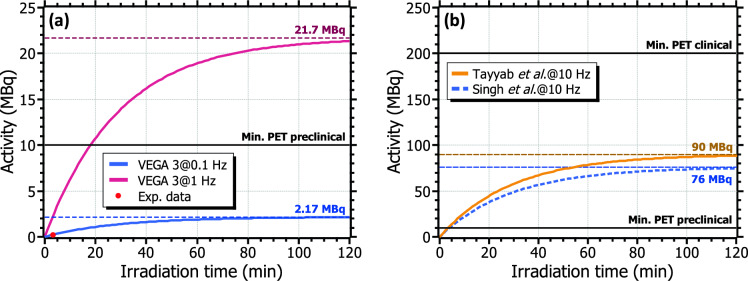
(**a**) Variation of the produced $^{11}$C activity in terms of the irradiation time at VEGA-3 for the experimental (0.1 Hz) and the nominal (1 Hz) repetition rates, depicted by the blue and magenta curves, respectively. The red dot indicates the maximum activity achieved in the experiment. (**b**) Estimated $^{11}$C activity at compact TW-class laser systems operating at 10 Hz, based on the results reported in M. Tayyab *et al.*^24^ (orange) and P. K. Singh *et al*.^33^ (blue). The minimum activity required for preclinical and clinical PET imaging is also depicted in the two figures.

**Figure 7:**
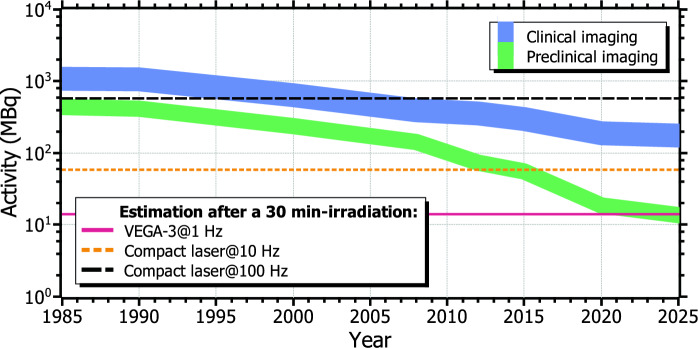
Inferred evolution of the typical activity used in PET for clinical (blue) and preclinical (green) imaging from data found in the literature^12–15,34^. The estimated $^{11}$C activity after a 30 min irradiation at VEGA-3 operating at 1 Hz and at a compact laser operating at 10 Hz are represented by the magenta solid line and the orange dotted line, respectively. The same case at 100 Hz is depicted by the black dashed line. The estimations for a compact laser were calculated from the single-shot experimental data reported in Tayyab *et al.*^24^.

These results can be extrapolated to longer operation times and higher repetition rates. Fig. 6a depicts the total induced activity over time during the irradiation, estimated using Eq. 4 and considering the production per shot of the experimental series with the highest pulse energy, 25 J (blue curve). Saturation—secular equilibrium— occurs after about 2 h of continuous irradiation. The maximum achievable activity in secular equilibrium is equal to the production rate $R = P\cdot f_N$, where $f_N$ denotes the laser pulse rate and *P* refers to the amount of $^{11}$C nuclei per shot. The maximum activity of $^{11}$C after reaching this regime is around 2.2 MBq at 25 J-pulse energy and 0.1 Hz. Increasing the repetition rate of up to the nominal value of the system, 1 Hz, would escalate this activity to almost 22 MBq (magenta curve in Fig. 6a). Consequently, a peak activity of 1.2 MBq would be induced after the completion of one of the eight sectors of the target wheel, i.e., 101 shots (nearly 2 minutes of operation), and 14 MBq after a 30 min-irradiation. Therefore, operating under these conditions would lead to $^{11}$C activities matching the range of PET preclinical imaging (10–30 MBq). In addition, further optimization of the laser system may increase the pulse intensity on target and would produce higher activities per shot.

A pertinent comparison can be made with respect to compact, table-top laser systems, which namely can operate at multi-Hz rates at the cost of less energetic pulses (0.1–3 J). Recently published measurements and predictions at these systems give single-shot $^{11}$C activities of the same order as the results presented in this work: between 1–6 kBq for compact lasers (shown in Table 1) and around 12 kBq at VEGA-3. While the production at a PW-class laser is expected to be much higher, the similarity of these results arises from the non-optimized laser parameters within the experiment. Consequently, at 1 Hz-repetition rate similar production levels can be assumed at compact lasers, while for a 10 Hz-operation these values would escalate up to several tens to almost a few hundreds of MBq in secular equilibrium. An estimate of the typical attainable activity can be performed by taking into consideration recent publications on laser-driven proton acceleration and radioisotope production at optimized compact lasers in literature. In P. K. Singh *et al*.^33^, proton bunches of up to 9 MeV were obtained by focusing laser pulses of 3 J and 30 fs on thin foils. A simple calculation, detailed in the *Methods* section, results in 4.4 kBq of $^{11}$C per laser shot considering the experimental spectrum reported in the publication (see Fig. 9). This estimated production is compatible with the direct measurement reported in M. Tayyab *et al*.^24^, of 5.2 kBq in a single shot. Operating at 10 Hz, it would correspond to respective maximum activities of 76 and 90 MBq, as depicted by the colored curves in Fig. 6b. It can be seen in the figure that these values exceed the minimum required for PET preclinical imaging, while in fact they are in the same order but do not reach PET clinical requirements.

Laser systems able to operate at even higher repetition rates, e.g. 100 Hz, are nowadays feasible^35–37^. Recently, some target designs capable of functioning at 100 Hz-repetition rate have been developed^38^, showing a great shot-to-shot stability and proton energies almost in the range of nuclear activation. Through further optimization, such systems could be used for radioisotope production in the context of medical imaging. The attainable activities would be then escalated by a factor of between 10 and 100, thus reaching the requirements for clinical PET imaging within shorter operation times. Moreover, the lower limit for the activity required in PET clinical and preclinical imaging is gradually decreasing year-to-year, as the imaging technologies and procedures become more efficient. Therefore, they might be attainable soon at laser systems similar to those referred in this work. In that respect, Fig. 7 shows the evolution of the minimum required activity in PET imaging over the last decades. The $^{11}$C activity after a irradiation of 30 min for VEGA-3 operating at 1 Hz and the estimation for a compact laser are depicted by the magenta solid line and the orange dotted line, respectively. The black dashed line corresponds to the same calculation for 100 Hz. As seen, preclinical imaging levels are in principle nowadays reachable at both TW- and PW-class systems. Regarding PET clinical imaging, besides being only achievable at potential 100 Hz-operation, current trends may reduce this limit down to repetition rates of state-of-the-art, standard laser systems.

## Conclusions and future perspectives

In conclusion, we have evaluated the use of laser-driven accelerators as an alternative technology for the production of radioisotopes of interest in PET medical imaging. Albeit current literature on this topic is limited to single-shot operation, this paper focuses on reaching sufficient activities for preclinical PET by accumulating a large number of shots, i.e., through multi-shot irradiation series. In this way, the production of $^{11}$C was successfully achieved for the first time using a PW-class laser system functioning in continuous operation.

As a first result, in this paper we report the systematic acceleration of protons of 10–14 MeV over long multi-shot series at repetition rates ranging from 0.1 to 1 Hz. Such operation constitutes a novel result in the context of laser-driven ion acceleration at a PW-class laser system. The proton cut-off energies, measured in several series of more than 101 shots using laser pulses up to 25 J, exhibit great stability and repeatability within 10%.

The irradiation of a boron target with these protons was performed over several multi-shot series, in order to produce $^{11}$C via the proton-induced $^{11}$B(p,n)$^{11}$C nuclear reaction. A peak activity of 234 kBq was reached within the experiment for a single series of 20 shots at 0.1 Hz and at the maximum laser energy. The corresponding activity per shot at the same series was 12.4 kBq. Increasing the irradiation time up to the completion of the entire target capacity—808 shots, which at 0.1 Hz would be around 2 h— would lead to 2.2 MBq, almost in the range of PET preclinical imaging. Moreover, achieving operation at 1 Hz, even for lower pulse energies, could increase the obtained activity a factor of 10, up to this range.

The repetition rate of the laser system results to be a highly influential factor for radioisotope production. It has been shown that this parameter directly affects the production rate and hence, the maximum achievable activity in secular equilibrium. Standard, present compact laser systems can deliver laser pulses at repetition rates between 1 to 10 Hz. The estimations performed in this work suggest that these lasers, operating at their maximum capability, would be able to reach activities close to PET clinical levels —hundreds of MBq— within long irradiation times. Furthermore, the next generation of compact lasers, already being developed, would be able to reach pulse rates up to 100 Hz and thus improve the reliability of laser-driven accelerators as a cost-effective alternative for radioisotope production.

## Methods

### Experimental details

A Thomson parabola spectrometer^39^ was used to measure the energy spectrum and the cut-off energy of the laser-accelerated protons during the irradiation series. A pinhole of diameter 200 µm was placed at the entrance of the TPS to collimate the ion beam. The active detector was a micro-channel plate (MCP) from Hamamatsu (F2813-12P) located at a fixed distance from the pinhole entrance. The images of the MCP were retrieved by means of a CCD camera, located behind the TPS.

Due to the high amount of energy deposited on target from the laser pulses, a huge electromagnetic pulse (EMP) was produced at the laser-solid interaction. To handle these EMPs and prevent a malfunctioning of the target assembly, a relay system was designed and implemented. On activation, the relays were programmed to disable the wired connections of the moving stages, so the EMPs could not propagate through them. Both the positioning of the targets at the laser focus and the action of these relays were synchronized with the laser pulses by means of the laser trigger signals. Higher laser pulse energies led to more intense EMPs, which in turn led to longer recovery times of the target assembly. Thus, the final operation rate was limited by the energy of the laser pulses, being 0.1 Hz for the maximum 25 J, 0.2 Hz for 20 J, and 1 Hz for 12 J.

Regarding the detection system for the measurement of the $^{11}$C activity, each detector was composed of a CsI(Tl) cylindrical scintillator attached to a Hamamatsu R6231 photo-multiplier (PMT) with a Scionix silicon pad. CsI(Tl) inorganic crystals have a density (4.5 g/cm$^3$) and a relative high effective atomic number ($Z_{eff}\approx$54) that grant a high detection response to photons of energies between 0.05 and 8 MeV. The scintillators had a diameter of 50.8 mm and 40 mm-length, and were covered by a plastic case of 0.5 mm-thick to reduce external light noise. The PMTs were biased using a NHQ 225M high-voltage power supply. Both detectors were connected to separate channels of a CAEN N5668B spectroscopic amplifier to shape and discriminate the retrieved signals. The output signals were digitalized by means of a CAEN DT5781 multi-channel analyzer (MCA), and sent to a PC for their visualization and analysis. In order to increase the detector efficiency, an aluminium plate of 2 mm-thickness was placed at the front face of each CsI(Tl) detector to induce the annihilation of the positrons escaping from the boron target. Positrons from the decay of $^{11}$C are emitted with an average energy of 385.7 keV and a maximum of 960.4 keV. The projected range of $\sim$1 MeV electrons or positrons inside aluminium is about 1.9 mm, as tabulated in the nist database^40^. Hence, the chosen thickness for the aluminium plates was sufficient to slow positrons down to the energy of the annihilation cross-section peak, about 100 keV, without attenuating the resulting photons.

In order to obtain a precise and discriminated event measurement of the decay of the $^{11}$C nuclei, the acquisition of the two CsI(Tl) detectors was configured in temporal coincidence. In this way, only photons reaching both detectors during a specific time window were registered. For the $\beta ^+$ decay of $^{11}$C, these events correspond to the correlated, back-to-back photons emitted in positron annihilation. Coincidence measurements were relevant in the experiment because other sources of gamma photons of similar energies could be generated by the laser-driven protons on the boron target. The deexcitation of beryllium-7, produced via the $^{10}$B(p,$\alpha$)$^7$Be reaction, emits a gamma photon of 477 keV which could not be resolved from the 511 keV photopeak due to the energy resolution of the CsI(Tl) detectors.

### Calibration of the detection system

**Figure 8:**
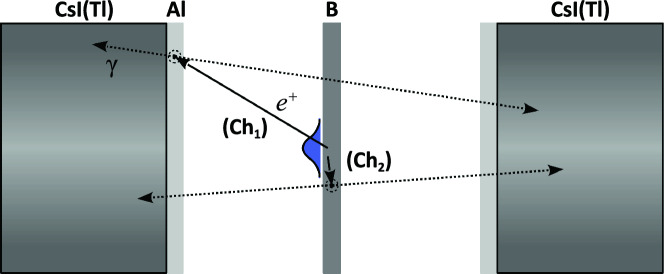
Schematic of the experimental coincidence configuration, depicting the two possible channels for the detection of the 511 keV photons from the positron annihilation. The width of the boron disk and the aluminium plates were 2 mm.

The complete detection setup for the characterization of the activity induced in the boron disk is depicted in Fig. 8. Concerning the calibration of the detection system, the generation and the detection of the 511 keV $\gamma$-photons can be separated into two main channels. Through the first one (*Ch*$_1$), a certain fraction of the $\beta ^+$ positrons will escape from the boron target without annihilating. Some of these positrons will reach the aluminium moderator in front of the detector and will decay there. Then, for the coincidence detection of the two back-to-back photons, each of them must be emitted from the aluminium plate in the direction of each detector. For the second detection channel (*Ch*$_2$), the positron is instead annihilated within the boron target and each photon is symmetrically emitted towards the detectors.

Taking these considerations, the geometrical efficiency of the detection system was estimated through Monte Carlo simulations using the EnsarROOT code^41^. Both detectors, as well as an extensive radioactive source of 511 keV, were defined for various source-detector distances following the experimental configuration. The activity distribution of the source was assumed Gaussian-like, given the Gaussian angular distribution of laser-accelerated protons in terms of their energy^42^, and estimated using a matlab code (detailed in *Activity estimations* within this section). Thus, the geometric efficiency was calculated for each of the two described detection channels. Each channel was weighted taking into consideration the number of induced activations in terms of the depth inside the target, i.e., the positron emission distribution, and the projected range of positrons in boron. The final estimated value was $\varepsilon _g^c$ = 0.31 ± 0.02. On the other hand, the photopeak intrinsic efficiency of the detectors at 511 keV was empirically determined by studying the response of the detectors to a collection of calibration radioactive sources with photopeaks within a range of several hundreds of keV, following the standard procedure. From both the geometrical factor and the intrinsic efficiency of the detectors at 511 keV, $\varepsilon _i$ = 0.35 ± 0.03, the total photopeak efficiency in coincidence configuration can be obtained via

1 $$\begin{aligned} \varepsilon _t^c = \varepsilon _g^c \cdot \varepsilon _i^2 \end{aligned}$$

In this way, the total efficiency at 511 keV resulted in $\varepsilon _t^c$ = 0.038 ± 0.007.

### Activity measurements

Given a certain number of counts $\Delta N$ registered in the detection system over a time interval $\Delta t$, the averaged activity *A* of the source within that time can be calculated via

2 $$\begin{aligned} A = \frac{\Delta N}{\Delta t}\cdot \frac{1}{\varepsilon _t^c \cdot I_{\gamma } \cdot BR} \end{aligned}$$

where $\varepsilon _t^c$ refers to the total efficiency in coincidence configuration of the detection system, and $I_\gamma$ and *BR* are the emission intensity and branching ratio, respectively. For the $\beta ^+$ decay of the $^{11}$C nuclei, these two parameters can be regarded as $\sim$1. By concatenating multiple measurements within sufficient time, the activity curve of the radioactive source can be characterized through the well-known radioactive decay law

3 $$\begin{aligned} A(t) = A_0 \exp (-\lambda _c t) \end{aligned}$$

being $A_0$ the initial activity—achieved immediately after the last laser shot—, and $\lambda _c$ the disintegration constant, which serves as univocal identification of the decaying radioisotope. During the laser irradiation, the radioactive decay is compensated by new nuclear activations and thus Eq. 3 can be updated to

4 $$\begin{aligned} N(t) = A(t)/\lambda _c = R (1 - \exp (-\lambda _c t))/\lambda _c \end{aligned}$$

In this expression, *N*(*t*) is the number of nuclei at a given time and *R* corresponds to the production rate. If the production rate is initially much greater than its decay rate, the production tends to a maximum value in the formally called *secular equilibrium* regime. This leads to a maximum achievable production after a certain operation time. Experimentally, the typical picture is that the amount of nuclei grows according to Eq. 4 during the production phase. After the production stops, the achieved activity level continues its decay following Eq. 3. The identification of the produced radioisotopes can be then performed in that phase through the determination of their characteristic emission spectrum and their half-life. For pulsed production sources, Eq. 4 can be utilized considering $R = P\cdot f_N$, being $f_N$ the laser pulse rate and *P* the amount of $^{11}$C nuclei per shot. This approximation is valid for high repetition rates or a low number of shots, where either the time-integrated production rate is constant or the accumulated error is small, respectively.

### Activity estimations

**Figure 9:**
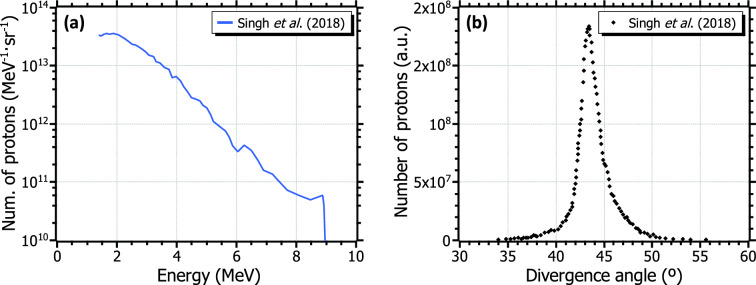
Experimental proton energy spectrum (**a**) and simulated angular divergence (**b**) used for the estimation of the induced activity on target. Data are taken from Singh *et al*.^33^.

The induced activity distribution at the irradiated target was estimated by means of a matlab code. Through this calculation, a proton beam of given energy spectrum and angular distribution impinges on a boron target. For each iteration within a defined depth step inside the target, the number of induced nuclear reactions and the energy loss are calculated for each angular and energy bins. The cross-section of the $^{11}$B(p,n)$^{11}$C nuclear reaction and the stopping power of proton in boron were taken from M.L. Firouzbakht *et al*.^10^ (see Fig. 1) and the srim software^43^, respectively. Particles with energies below the energy threshold of the reaction are removed from the calculation for subsequent iterations.

The thickness of the activation target is enough to stop protons up to 15 MeV, i.e., slightly thicker than 1.3 mm. Thus, all incidental particles interact or are completely stopped inside the target. From the resulting total production—i.e., the activity at a single shot— the final activity after a series of shots is calculated from Eq. 4 in terms of the irradiation time and the repetition rate of the laser system. The estimations performed within this work were performed considering the data on laser-driven proton acceleration reported in P. K. Singh *et al*.^33^. Fig. 9 shows the experimental energy spectra (a) and the simulated angular divergence (b) retrieved from this publication to perform the calculations. The latter was normalized and fitted to a Gaussian distribution, resulting in a FWHM (Full Width at Half Maximum) of 4.5$^\circ$.

## Data Availability

The data generated and analyzed during the current work are available from the corresponding author on reasonable request.
